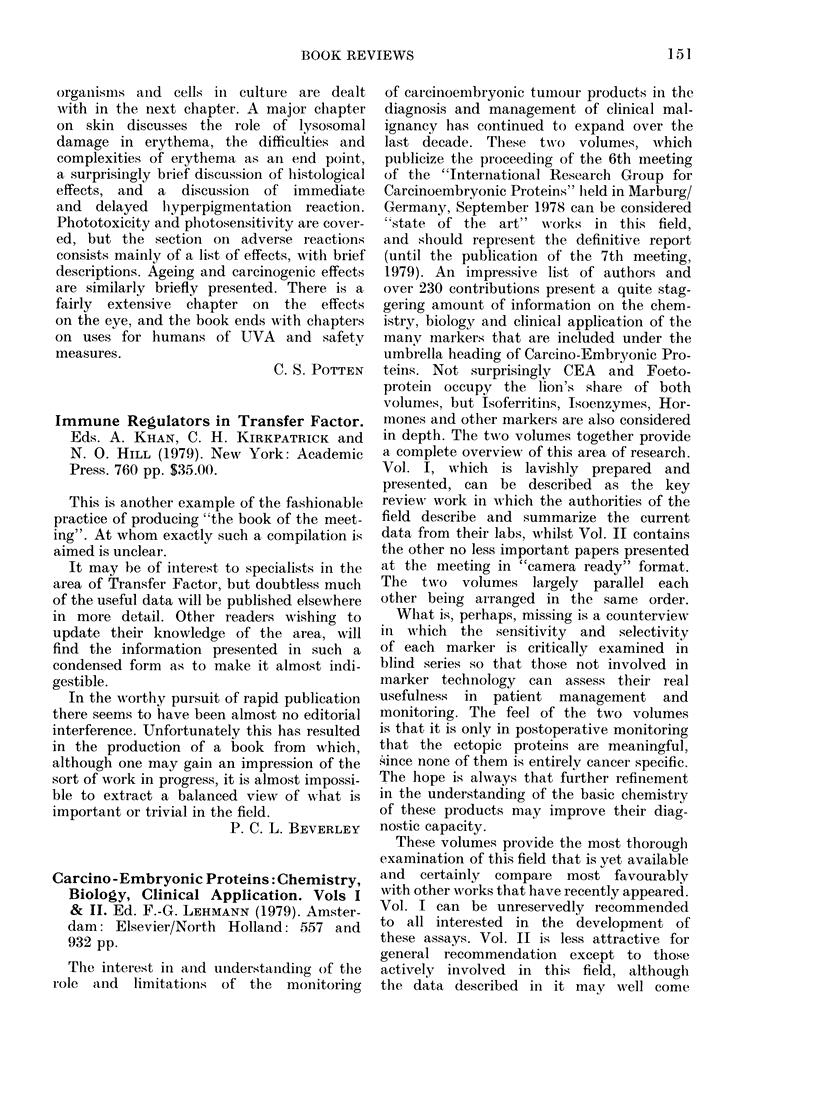# Immune Regulators in Transfer Factor

**Published:** 1980-01

**Authors:** P. C. L. Beverley


**Immune Regulators in Transfer Factor.** Eds. A. KHAN, C. H. KIRKPATRICK and N. O. HILL (1979). New York: Academic Press. 760 pp. $35.00.

This is another example of the fashionable practice of producing "the book of the meeting". At whom exactly such a compilation is aimed is unclear.

It may be of interest to specialists in the area of Transfer Factor, but doubtless much of the useful data will be published elsewhere in more detail. Other readers wishing to update their knowledge of the area, will find the information presented in such a condensed form as to make it almost indigestible.

In the worthy pursuit of rapid publication there seems to have been almost no editorial interference. Unfortunately this has resulted in the production of a book from which, although one may gain an impression of the sort of work in progress, it is almost impossible to extract a balanced view of what is important or trivial in the field.

P. C. L. BEVERLEY